# Examining the Role of Vasopressin in the Modulation of Parental and Sexual Behaviors

**DOI:** 10.3389/fpsyt.2015.00130

**Published:** 2015-09-22

**Authors:** Josi Maria Zimmermann-Peruzatto, Virgínia Meneghini Lazzari, Ana Carolina de Moura, Silvana Almeida, Márcia Giovenardi

**Affiliations:** ^1^Programa de Pós-Graduação em Fisiologia, Instituto de Ciências Básicas da Saúde (ICBS), Universidade Federal do Rio Grande do Sul (UFRGS), Porto Alegre, Brazil; ^2^Programa de Pós-Graduação em Ciências da Saúde, Universidade Federal de Ciências da Saúde de Porto Alegre (UFCSPA), Porto Alegre, Brazil

**Keywords:** evolutionary lineage VP, vasopressin-like, V_1a_ receptor, V_1b_ receptor, social behaviors

## Abstract

Vasopressin (VP) and VP-like neuropeptides are evolutionarily stable peptides found in all vertebrate species. In non-mammalian vertebrates, vasotocin (VT) plays a role similar to mammalian VP, whereas mesotocin and isotocin are functionally similar to mammalian oxytocin (OT). Here, we review the involvement of VP in brain circuits, synaptic plasticity, evolution, and function, highlighting the role of VP in social behavior. In all studied species, VP is encoded on chromosome 20p13, and in mammals, VP is produced in specific hypothalamic nuclei and released by the posterior pituitary. The role of VP is mediated by the stimulation of the V_1a_, V_1b_, and V_2_ receptors as well as the oxytocinergic and purinergic receptors. VT and VP functions are usually related to osmotic and cardiovascular homeostasis when acting peripherally. However, these neuropeptides are also critically involved in the central modulation of social behavior displays, such as pairing recognition, pair-bonding, social memory, sexual behavior, parental care, and maternal and aggressive behavior. Evidence suggests that these effects are primarily mediated by V_1a_ receptor in specific brain circuits that provide important information for the onset and control of social behaviors in normal and pathological conditions.

## General Aspects

Vasopressin-like (VP-like) and oxytocin-like (OT-like) peptides have been isolated from invertebrates and vertebrates in more than 100 species (1). Approximately 700 million years ago, the ancestral gene encoding the precursor protein diverged between the invertebrate and vertebrate families (2). Generally, all vertebrate species express a VP-like and OT-like peptide.

The lineage of VP-like peptides is evolutionarily stable. In fishes, amphibians, reptiles, and birds, vasotocin (VT) shares similar roles with mammalian arginine vasopressin (VP), whereas mesotocin and teleost isotocin are functionally similar to mammalian oxytocin (OT) (3).

The gene expression and regulation of these peptides is conserved among vertebrates (3). The chemical structure of VP and OT differs by only two of nine amino acid residues (4, 5). The VP/OT superfamily can be traced back to different types of invertebrates, such as annelids and mollusks (6). There is a homology of 80% between VP and OT, but these neuropeptides have distinct physiological activities. The receptors for VP-like and OT-like peptides have been described in invertebrates (6) and vertebrates (7).

Both VP and OT are produced in the hypothalamus, released in the neurohypophysis, and distributed throughout the brain (8, 9). In 1895, the vasopressor effect of the neurohypophyseal extract was attributed to the neurohypophysis gland (10). However, two decades later, the antidiuretic effect of the pituitary extract was demonstrated (11). The isolation of VP in the fifties confirmed that the same neuropeptide is synthesized in the neurohypophysis gland and possesses antidiuretic and vasopressor effects (12).

This review focuses on the involvement of VP in brain circuits, synaptic plasticity, evolution, and function, highlighting the role of VP in parental and sexual behaviors.

## Vasopressin and Vasopressin-Like Peptides

### Gene structure

Nucleotide sequences encoding the VP and OT hormones are highly homologous. In all species, VP and OT are located on the same chromosome, 20p13, but encoded by different genes separated by a segment of DNA only 12 kb long (13). The similarity in the intron–exon structures of the two genes and opposite orientation suggest recent gene duplication (14).

### Synthesis and release

Vasopressin is a nonapeptide with a disulfide bridge between two cysteine amino acids and is synthesized in a smaller amount by parvocellular neurons, primarily by magnocellular neurons of the hypothalamus in paraventricular nucleus (PVN) and supraoptic nucleus (SON) (15). These nuclei send axons to the neuro pituitary along the supraoptic–hypophyseal tract. In a prohormone state, VP migrates along the supraoptic–hypophyseal tract to the neurohypophysis gland, where it is released into circulation (16).

The PVN and SON receive afferent nerve impulses from receptors in the left atrium, aortic arch, and carotid sinuses via the vagus nerve. PVN and SON receive osmotic input from the lamina terminalis, which is excluded from the blood–brain barrier and is thus affected by systemic osmolality. Furthermore, Holmes et al. (16) suggested that in the rat brain, extrahypothalamic structures, such as the bed nucleus of the stria terminalis (BNST), the medial amygdala, nucleus of the locus coeruleus, hippocampus, and choroid plexus, in addition to the hypothalamus, are able to synthesize VP.

The anterior pituitary gland also releases VP but in smaller quantities. VP can activate the hypothalamic–pituitary–adrenal axis by stimulating the V_1b_ receptor (V_1b_R) and controlling the liberation of adrenocorticotropic hormone (ACTH) (17), whereas the V_1a_ receptor (V_1a_R) controls the synthesis and release of cortisol in the adrenal cortex (18).

### Vasopressin receptors

The functions of VP are modulated by stimulation of G-protein-coupled receptors (GPRCs) from each independent tissue. They are classified as V_1a_R, V_1b_R (also known as V_3_R), V_2_R, oxytocinergic (OTR), and P_2_ purinergic (P_2_R) receptors (19, 20). In mammals, the VPRs are widely distributed in the brain (21, 22).

Vasopressin can bind to all of these receptors but not with the same affinity. The receptors, which use G-proteins as transducer signals across the cell membranes, have seven hydrophobic transmembrane domains, four extracellular domains, and four intracellular domains (23). Neurotransmitters, hormones, and chemokines indicate the courses of VP action, whereas local mediators signal to the four main G-protein families to regulate metabolic enzymes, ion channels, and transcriptional regulators. The different types of VPR extracellular signals refer to specific G-proteins. Several important hormones interact with the G_i_ pathway, which is characterized by inhibition of adenylyl cyclase (24).

Agonist of VPR is a substance that initiates a physiological response through specific interactions with G-protein-coupled receptor kinases and protein kinase C present in the carboxyl termini of the receptors (25). The VP signal is transmitted through guanine nucleotide-binding proteins (G-proteins) (26), such as G_s_ and G_q/11_ subtypes (24).

#### V_1a_R Receptor

V_1a_R is primarily found on vascular smooth muscle and causes vasoconstriction by an increase in intracellular calcium via the phosphatidyl-inositol-bisphosphonate cascade. Studies in rats have shown that V_1a_R is also located in the brain, myocardium, gonads, cervical ganglion, liver, blood vessels, kidney, spleen, renal medulla, and platelets (20–22, 27–29); however, the physiologic roles of VP remain unknown in many tissues.

#### V_1b_R Receptor

V_1b_R is localized in pituitary gland, olfactory bulb, septum, hippocampus, pancreatic beta cells, and adrenal medulla and induces the release of hormones (20, 30–32). The phylogenetic analysis showed that V_1b_R diverged early from the V_1a_R sequences and presented the closest relationship with the OTR (31). V_1b_R is highly expressed in the anterior pituitary where it is thought to play a role in costimulating the neuroendocrine response to stress (33). The VP causes secretion of ACTH, which is important for the induction and phenotype maintenance of ACTH-secreting tumors mediated through G_s_, G_i_, and G_q/11_ (34). Studies have shown that V_1b_R gene expression may thus be a marker of the corticotroph phenotype and can be used to help shed light on the pathophysiological mechanism of ectopic ACTH syndrome (30, 35).

#### V_2_ Receptor

V_2_R is located on vascular smooth muscle cells, vascular endothelium, and the collecting ducts of the renal medulla (20). The hydro-osmotic or antidiuretic effect of VP occurs via activation of V_2_R (36, 37). VP adjusts water homeostasis regulation of the fast shuttling of aquaporin 2 to the cell surface and stimulates the synthesis of mRNA encoding this protein (38, 39).

Phillips et al. (28) evaluated V_1_Rs and V_2_R binding sites *in vitro* using selective radioligands and demonstrated that there was no vasoconstrictor activity of V_2_R in the endothelium, liver, brain, spinal cord, sympathetic ganglia, heart, or vascular smooth muscle. In this study, specific binding was only identified in the kidney, which is consistent with the known distribution of antidiuretic V_2_Rs on renal collecting tubules (28).

#### Other Receptors

Both VP and OT can bind with OTR but not with the same affinity. OTRs are coupled to G_q/11_ class binding proteins, which stimulate phospholipase C activity (34).

P_2_ purinergic receptors also belong to the seven-transmembrane domain GPCR superfamily. Its role was confirmed in cardiac endothelium because VP exhibited effects through activation of P_2_Rs (40).

## Vasopressin and Synaptic Plasticity

Vasotocin/vasopressin neuropeptides affect several sex-typical and species-specific behaviors and produce an integrational neural substrate for the dynamic regulation of these behaviors via endocrine and sensory stimuli. Different types of social behaviors are influenced by VT and VP, influencing the objectives of many neuroanatomical studies of VT/VP distribution and central nervous system (CNS) targets (4, 41, 42). There is a sexually dimorphic vasopressinergic extrahypothalamic network that plays a key role in the modulation of behavior by VP (43), and sex differences in VP distribution were first demonstrated in rats (44).

In vertebrates, behaviors such as reproductive, smell recognition, social communication, pair-bonding, parental care, and aggressive behavior are also modulated by VT/VP (41, 45, 46). In different species of vertebrates, both central and peripheral VT/VP administration stimulate spawning behavior, phonotaxis, sexual receptivity, lordosis, courtship, and mating (4, 41, 47).

In the rat brain, V_1a_R is believed to play the predominant role in regulating behavior. V_1a_R is found in neural networks involved in responses related to social behavior and exhibits considerable plasticity (45, 46, 48). V_1a_R is expressed in olfactory bulb, hippocampal dentate gyrus, cerebellum, septal nuclei, accumbens nucleus, arcuate nuclei, suprachiasmatic nuclei, and periventricular nuclei and the lateral hypothalamic area, parvocellular paraventricular and anteroventral nucleus of the thalamus, circumventricular organs including the pineal, and the subfornical organ (49, 50). Further, V_1b_R also participates in the neural regulation of social behaviors (51), but has received much less attention due to a lack of specific drugs (52, 53). V_1b_R transcripts and immunoreactive cell bodies are localized to the cerebellum, cerebral cortex, hippocampus, olfactory bulb (including in the area periglomerular), PVN, piriform cortex layer II, red nucleus, septum, and suprachiasmatic nucleus (54–61).

Vasopressinergic as well as oxytocinergic systems can be modulated by circulating gonadal steroids and together are involved in behavioral regulation (4, 41, 62, 63). Sex hormones affect the quantity and localization of the VT/VP receptors in the brain (64). Previous studies (64, 65) have shown that behavioral responses, such as courtship behavior in newts, aggression in hamsters, and parental behaviors in voles (66), depend on VT/VP and the presence of gonadal steroids.

### Role of vasopressin in parental and sexual behavior

The ability of an animal to recognize intra- and interspecific individuals is essential for all complex relationships. Several studies (5, 46, 63, 67–71) have shown that VP participates in the modulation of non-social and social behaviors.

In this sense, vasopressinergic neurons play an important role in coding information from social contact (47). In rodents, social information is primarily mediated by the exchange of olfactory information, and there is evidence that VP signaling is important in brain areas where olfactory information is processed. Wacker et al. (51) described populations of vasopressinergic neurons in the main and accessory olfactory bulbs and anterior olfactory nucleus that are involved in processing social odor cues. Pharmacological studies have shown that VP administration improves social recognition in both sexes (58, 63). If applied bilaterally in the olfactory bulbs, extending the memory retention interval for the recognition of male rat odor is extended (72).

In rodents, intracerebroventricular microinjections of VP agonists facilitate social memory and can significantly extend social recognition and memory consolidation for as much as 120 min (73, 74). V_1a_R antagonists produce marked effects on learning, memory, and social behaviors (59, 63). Intracerebroventricular and intraseptal microinjection of V_1a_R antagonists block social recognition, and VP microinjection can rescue deficits in social recognition in Brattleboro rats that lack VP (74–77).

Bielsky et al. (45) demonstrated that V_1a_R knockout mice (V_1a_RKO) display an enormous deficit in social recognition, providing strong evidence that V_1a_R is essential for this action. Similarly, V_1b_R knockout mice (V_1b_RKO) also presented deficits in the social memory test (52, 53). However, the increased V_1a_R expression in the lateral septum (LS) facilitated social behavior (78).

Among other social behaviors, the parental behavior exerts an essential role in the behavioral development of offspring (79). Maternal care is best studied; however, pup-directed positive behaviors, such as retrieval and kyphosis (huddling), are exhibited by no-parturient animals and are characteristics of socially monogamous or cooperative breeding species. Male parental care has been primarily studied in relation to the vasopressinergic system, which is sexually dimorphic and androgen-dependent because testosterone promotes VP synthesis (79).

Paternal care is highly demonstrated by prairie vole males (80, 81). Lim and Young (82) demonstrated that the infusion of VP (0.1 ng) directly into the LS of these animals can enhance licking/grooming (LG) behavior toward pups and that this behavior is blocked by the use of a V_1a_R antagonist (80). The father LG behaviors and retrieving the pups exert a pivotal role on the development of the VP systems and aggressive behavior of their adult offspring (79). In adult male rats, LG behaviors increase the levels of V_1a_R binding within the amygdale nucleus (46).

Besides the parental behavior, the VP system is also an important mediator of maternal behavior. In the peripartum and lactation periods, there is an increase in the expression of mRNA, receptors, and density/binding of VP in the maternal brain. This increase contributes to the adaptations that develop in the female maternal care. Previous studies (83–85) have already suggested a role for VP facilitating maternal care, but more recent studies showed substantial evidence of this neuropeptide facilitating maternal behavior [for review, see Bosch and Newman (86)]. Furthermore, in females, V_1a_R density was significantly correlated with postpartum LG of the offspring (87).

Most studies are based on infusion of VP and V_1a_R antagonists in specific areas of the CNS participating in the neural circuitry of maternal behavior and thus demonstrating the modulation of VP in this behavior [for review, see Ref. (86, 88–91)].

Moreover, previous studies (92, 93) with HAB (high anxiety-related behavior) and LAB (low anxiety-related behavior) dams also reinforce that central VP modulates the maternal behavior because the blocking of V_1a_R by repeated acute intracerebroventricular administration of a selective antagonist promoved decrease arched back nursing and the time the dam spent with the pups in HAB dams.

In conjunction with these findings, the involvement of V_1b_R in the modulation of maternal behavior was demonstrated in V_1b_RKO mice (94), which showed that lactating females who received the V_1b_R antagonist in the lateral ventricle decreased the nursing and interaction with their pups.

In addition to maternal care, lactating rats exhibit aggressive behavior that is observed during the first two postpartum weeks and which aims to protect the offspring against a potentially dangerous intruder (95, 96). The role of VP in aggression has received attention, and previous studies (86, 88, 89, 91, 97–100) provide substantial evidence for VP promoting maternal aggressive behavior. Neuroanatomical studies with multiparous rats revealed significant increases in V_1a_R mRNA expression in the amygdala, SON, and LS in females on the fifth postpartum day when compared with primiparous rats (101, 102).

Bosch and Neumann (90) demonstrated that microinjection of a selective V_1a_R antagonist bilaterally into the BNST reduced maternal aggression (91) and the VP within the central nucleus of the amygdala (CeA) was positively correlated with the increased offensive behavior (90). Furthermore, other study by Bosch and Neumann (92) reported that in HAB rats VP promotes maternal aggression, and Lonstein and Gammie (103) showed the increased expression of the VP gene in the PVN. On the other hand, studies with Sprague-Dawley lactating rats showed different results, as the intracerebroventricular infusions of VP reduced maternal aggression, while treatments with an V_1a_R antagonist increased maternal aggression during early lactation (88).

Vasopressin-deficient Brattleboro rats exhibit reduced aggressive maternal behavior and reduced attacks in males without sexual experience against intruders (99). In other study on female pregnancy (51, 104), increasing V_1a_R levels in the PVN, CeA, and LS were positively correlated with aggressive behavior. The fluctuations observed in the OTR and V_1a_R in important areas of the CNS appear to regulate maternal aggression during the peripartum period. In a pharmacological study, microinjection of a selective V_1a_R antagonist bilaterally into the BNST reduced maternal aggression behavior but did not alter maternal care (105).

Furthermore, VP may be a new target for studies on treatments involving V_1a_R antagonists or synthetic VP to promote maternal care or suppress aggression in lactating females exposed to chronic stress-associated disorders (98). Using a model of VP-deficient mothers, Fodor et al. (105) demonstrated that these rats have decreased LG behavior and act less depressive. Thus, they suggest that VP antagonists could be an option for future studies on postpartum depression; however, the possible side effects of maternal neglect require further investigation (90, 105).

Some findings suggest that V_1b_R might also be involved in the modulation of aggressive behavior in both females and males. V_1b_RKO lactating mice showed an increased latency and decreased number of attacks against intruders when compared with wild-type mothers (97). The defensive behavior was studied in V_1b_RKO male mice and increased social behavioral responses were observed (106). In support, V_1b_RKO male mice also had impaired attack behavior toward a conspecific (97).

The role of this nonapeptide in sexual behavior has also been described in the past decades, and previous studies showed that VT/VP modulates specific types of vocalization in rats and squirrel monkeys (107). VT/VP central administration enhances pair-bonding and smell-recognition behaviors as a key feature for the onset of sexual behavior (66, 107).

Since the 1980s, studies have described the importance of vasopressinergic projections in both sexes in relation to sexual behavior. In this context, Meyerson et al. (104) administered an antagonist of VPRs into the lateral ventricles of female Sprague-Dawley rats in the neonatal period; this treatment induced a persistent increase in VP content and facilitated female sexual behavior. This study provided functional and immunocytochemical evidence of the importance of VP in female sexual behavior. In the same decade, Södersten et al. (108) reported that intracerebroventricular injections of VP inhibit sexual behavior in receptive female Wistar rats. These findings were reinforced by Pedersen and Boccia (109), which suggested that VP influences ovarian steroid activation of female sexual behavior via interactions with OT.

In males, Bohus (110) observed that VP agonists reversed the decrease of sexual behavior after castration, but injections of VP into LS of males did not dramatically alter sexual behavior (111, 112). In the 1990s, the role of VP in the modulation of male sexual behavior was hypothesized to be due to the refractory period in rats and consequently be an inhibitor of sexual behavior in males (112). In 1993, Winslow et al. (66) reported that VP is necessary to partner preference formation in monogamous prairie vole. However, these studies commonly focused on differences in neurotransmitter systems in brain structure between sexes instead of the role of the neurotransmitter on sexual behavior. Knowledge about differences in cell density, neurotransmitter content, receptors distribution, and vasopressinergic projections in rats and voles between sexes does not necessarily cause sex differences in sexual behavior (113).

Knockout animals were also used to investigate the role of VP and its receptors in sexual behavior. V_1b_RKO mice exhibited deficits in social behaviors that require olfactory function, including aggression and social recognition, but these animals had normal sexual behavior (52). The BNST is a sexually dimorphic structure that can be involved in the control of male sexual behavior because males have more VP neurons and denser projections from this area and in the medial amygdaloid nucleus than females (114). However, studies about VP innervation have focused on female sexual behavior. VP innervation from the LS inhibits sexual behavior in females; thus, hypothetically, the higher levels of VP in males are correlated with less lordosis behavior (115).

In humans, VP has been reported as a selective enhancer of recognition of sexual cues in a behavioral task administered to males (116). Additionally, Argiolas and Melis (117) conducted an elegant review about the control of sexual behavior by neuropeptides in the species studied thus far, including rats, mice, monkeys, and humans (117), and describe VP as an ineffective neuropeptide on copulatory behavior in males but as an inhibitor of lordosis in female sexual behavior.

The role of VP in sexual behavior remains unclear. Controversial results can be explained by the different methods applied and the interactions of this peptide with others, which should always be considered.

In conclusion, the lineage for VP or VP-like neuropeptides is evolutionarily stable and present in all vertebrate species. The neural distribution of VP neurons and its projections and also the distribution of VPRs are widely investigated for more than 30 years. These extensive data collected shows that male and female have important differences in the VP distribution across the CNS. The role of VP in osmotic and cardiovascular homeostasis is well established in the literature; however, the neuropeptides VT/VP are also critically involved in the modulation of social behaviors in many species. In mammals, the parental and sexual behaviors are mediated by VT/VP system and mainly by V_1a_R in specific brain circuits that provide important information for the onset and control of these behaviors in normal and pathological conditions. Future investigations must be conducted to further elucidate the involvement of V_1b_R modulating social behaviors.

## Conflict of Interest Statement

The authors declare that the research was conducted in the absence of any commercial or financial relationships that could be construed as a potential conflict of interest.
